# Genome wide identification and expression analysis of gibberellin oxidase family genes in sweet potato and its two diploid relatives

**DOI:** 10.1038/s41598-026-37951-8

**Published:** 2026-02-01

**Authors:** Shanshan Zhang, Yongxiang Cao, Hui Yan, Ge Qing, Zhe Zhao, Wenbang Hou

**Affiliations:** 1 College of Agronomy, Henan University of Science and Technology, Luoyang, 471000 China; 2 Key Laboratory of Biology and Genetic Improvement of Sweet Potato, Ministry of Agriculture/Jiangsu Xuzhou Sweet Potato Research Center, Xuzhou Institute of Agricultural Sciences in Jiangsu Xuhuai District, Xuzhou, 221131 China

**Keywords:** Genetics, Plant sciences

## Abstract

As essential plant hormones, gibberellins (GAs) regulate various growth and development processes. GA oxidases (GAoxs), including the GA2ox, GA3ox, and GA20ox subgroups, play prominent roles in the synthesis and degradation of GA; however, a systematic analysis of GAoxs has not been reported in sweet potato. Here, we identified GAox genes based on a comparative genomic analysis in sweet potato (*I.batatas*) and its two diploid wild relatives *I. trifida* and *I. triloba*. A total of 71 GAox genes were identified, with 23 in sweet potato, 25 in *I. trifida* and 23 in *I. triloba*. These genes can be divided into four phylogenetic groups based on their protein sequences. Each subgroup had their own conserved motif combination. The prediction of *cis*-elements in the promoter region showed that the most numerous elements in the promoter regions were related to hormone response. GAox genes in sweet potato respond to both plant hormones and abiotic stresses; notably, gene expression and co-expression analysis implicated *ibGA2ox10* in tuber expansion, *ibGA20ox3*/*ibGA3ox5* in indole-3-acetic acid (IAA) and gibberellin (GA) synergy, *ibGA2ox5*/*ibGA3ox7* in gibberellin (GA) and paclobutrazol antagonism, and *ibGA20ox1*/*ibGA3ox8* in abiotic stress response.

**Supplementary Information:**

The online version contains supplementary material available at 10.1038/s41598-026-37951-8.

## Introduction

Gibberellins (GAs) are tetracyclic diterpene plant hormones that play diverse roles in regulating plant growth and development, such as seed germination, organ elongation and expansion through cell growth, transition from the seedling stage to the mature stage and from vegetative development to reproductive development^1^ The GAs comprise a large group of tetracyclic diterpenoid carboxylic acids with the *ent*-gibberellane (C20) or 20-nor-*ent*-gibberellane (C19) carbon skeleton^2^. To date, more than one hundred GAs have been identified in plants, fungi, and bacteria; however, only a small number can function as bioactive hormones, such as GA_1_, GA_3_, GA_4_ and GA_7_, and many non-bioactive GAs occur in plants as precursors of bioactive forms or deactivated metabolites^1^.

The biosynthesis of active GAs has been elucidated in many plant species, and it is a complex, multistep process with diverse intermediates. This process is initiated by geranylgeranyl diphosphate (GGPP), a common C20 precursor for diterpenoids^3^. Three different types of enzymes are needed in the synthesis of bioactive GAs in plants: terpene synthases (TPSs), cytochrome P450 monooxygenases (P450s), and 2-oxoglutarate-dependent dioxygenases (2ODDs). TPSs contain *ent*-copalyl diphosphate synthase (CPS) and *ent*-kaurene synthase (KS), which are involved in the conversion of GGPP to *ent*-kaurene, a tetracyclic hydrocarbon intermediate. These two types of TPSs are located in the plastids. Then, *ent*-kaurene is catalyzed to GA12 by two types of P450s, *ent*-kaurene oxidase (KO) and *ent*-kaurenoic acid oxidase (KAO)^4,5^. The last step catalyzed by 2ODDs is the synthesis of bioactive GAs. The GA oxidases (GAoxs) GA2ox, GA3ox, and GA20ox are all 2ODDs that belong to the 2-oxoglutarate and Fe (II)-dependent dioxygenase (2OGD) superfamily and are mainly responsible for GA synthesis and degradation in plants^6^. GAoxs can be divided into multiple small gene families. GA2ox contains C19-GA2oxs and C20-GA2oxs, with C19-GA as the preferred substrate of C19-GA2ox and C20-GA as that of C20-GA2ox^7^. GA3ox and GA20ox are the key enzymes involved in bioactive GA synthesis, which converts GA_53_ and GA_12_ to GA_1_ and GA_4_, respectively. In turn, GA2ox is involved in the degradation and inactivation of GAs (GA_1_ and GA_4_) and their precursors (GA_9_, GA_12_, GA_20_ and GA_53_)^8^.

The gibberellin oxidase (GAox) gene family, known to be critically involved in gibberellin biosynthesis and catabolism, has been characterized in multiple plant species including Arabidopsis^9^, rice^9^, and maize^10^. It has been demonstrated that GAox genes not only display distinct tissue-specific expression patterns but are also extensively involved in phytohormone signaling and abiotic stress responses, highlighting their functional versatility in plant development and adaptation^11^. For instance, photoperiod-dependent flowering is regulated by *GA20ox* through spatial–temporal expression in Arabidopsis^12^, while salt stress tolerance is enhanced by *OsGA2ox5* through modulation of stress-responsive pathways in rice^13^. Plant height is recognized as one of the most prominent agronomic traits controlled by GAox genes. The semidwarf architecture of rice has been shaped by the “Green Revolution” gene *sd1*, which encodes GA20ox, through reduced active gibberellin biosynthesis^14^. Similarly, desirable dwarfism is conferred by loss-of-function mutations in *OsGA3ox2* in ‘Xiao wei’ rice^15^. Furthermore, internode elongation has been consistently suppressed by *GA2ox* overexpression in Arabidopsis^16^ and rice^17^ through inactivation of bioactive GAs, confirming the conserved role of this gene family in height determination.

Sweet potato [*Ipomoea batatas* (L.) Lam] is globally recognized as an important staple root crop, ranking seventh in worldwide production and fourth in China^18^. Vine length control is considered crucial for optimizing the source–sink balance and achieving high yields in cultivation. Although gibberellin biosynthesis inhibitors such as paclobutrazol (PBZ) are widely used to suppress excessive shoot growth, their application is associated with potential chemical residues and environmental concerns. As a sustainable and precise alternative, the genetic dissection of GAox genes has been proposed; this approach has already been validated in the vine crop potato^19^. The sweet potato genome sequence has been anchored on 15 pseudochromosomes^20^. Although this genome has not been precisely assembled, it established the base for gene identification, cloning and functional analysis of cultivated sweet potato. In the present study, a total of 71 GAox genes were identified from the hexaploid sweet potato and its two diploid wild relatives. A comprehensive analysis was conducted, which included protein motif identification, gene structure characterization, cis-element annotation in promoters, and spatiotemporal expression profiling. In addition, transcriptional responses to various hormones (ABA, IAA, GA, 6-BA, and PBZ) and abiotic stresses (PEG-induced drought and NaCl) were assessed. This study provides a valuable genetic resource for elucidating GA metabolic pathways and lays a solid foundation for future research into the molecular mechanisms governing key agronomic traits in this vital crop.

## Results

### Identification of GA2ox, GA3ox and GA20ox in three Ipomoea genomes

Based on the protein sequences of GAox gene family members in *Arabidopsis thaliana*^9^, a BLAST search was performed to screen candidate genes in three *Ipomoea* genomes. *GA2ox*, *GA3ox* and *GA20ox* belong to the 2OGD superfamily, which has a conserved and characteristic motif, 2OG-FeII_Oxy (PF03171)^8^. Based on this criterion, we validated and purified the genes obtained from the BLAST results. Finally, a total of 23, 25 and 23 GAox genes were identified in the genomes of *I. batatas*, *I. trifida* and *I. triloba*, respectively (Table 1 and Supplementary Table S1). Among the 23 candidate genes identified in *I. batatas*, there were 11 *ibGA2ox*, eight *ibGA3ox*, and four *ibGA20ox* genes (Table 1). A total of 10 *itfGA2ox*, 12 *itfGA3ox*, and three *itfGA20ox* genes were identified in the *I. trifida* genome, with nine *itbGA2ox*, 11 *itbGA3ox*, and three *itbGA20ox* genes identified in the *I. triloba* genome (Supplementary Table S1), which indicated that the number of each type of GAox gene was equivalent across the three genomes.

**Table 1:** Putative GAox genes in *I. batatas*.

Gene symbol	Gene ID	Chr	Strand	Position	Peptide length (aa)	PI	MW (kDa)	Predicted localization
*ibGA2ox1*	*g5011.t1*	2	+	5,431,037–5,435,079	347	5.62	39.19	Cytoplasm
*ibGA2ox2*	*g5548.t1*	2	+	8,862,885–8,863,969	314	9.1	34.69	Cytoplasm
*ibGA2ox3*	*g13516.t1*	4	+	4,878,670–4,882,216	289	8.8	32.4	Cytoplasm
*ibGA2ox4*	*g26076.t1*	7	-	5,288,484–5,290,248	278	6.22	30.97	Cytoplasm
*ibGA2ox5*	*g26077.t1*	7	-	5,290,743–5,292,118	281	6.01	31.36	Cytoplasm
*ibGA2ox6*	*g34757.t1*	9	+	4,710,987–4,712,826	345	6.2	38.36	Cytoplasm
*ibGA2ox7*	*g37345.t1*	9	+	25,084,127–25,086,001	288	5.86	32.13	Cytoplasm
*ibGA2ox8*	*g43434.t1*	11	-	14,099,881–14,101,341	327	7.67	36.32	Cytoplasm
*ibGA2ox9*	*g54334.t1*	13	+	23,250,294–23,253,681	330	6.6	36.89	Cytoplasm
*ibGA2ox10*	*g56590.t1*	14	-	8,607,093–8,609,290	321	5.61	36.39	Cytoplasm
*ibGA2ox11*	*g59308.t1*	14	-	27,945,721–27,947,858	353	6.04	40.53	Cytoplasm
*ibGA3ox1*	*g26071.t1*	7	-	5,274,136–5,279,628	532	5.85	59.88	Cytoplasm
*ibGA3ox2*	*g26080.t1*	7	+	5,329,560–5,331,055	354	5.16	39.66	Cytoplasm
*ibGA3ox3*	*g26082.t1*	7	+	5,347,770–5,349,301	361	5.27	40.83	Cytoplasm
*ibGA3ox4*	*g26083.t1*	7	+	5,354,560–5,355,937	364	5.73	41.32	Cytoplasm
*ibGA3ox5*	*g26084.t1*	7	+	5,366,680–5,369,597	359	5.18	40.63	Cytoplasm
*ibGA3ox6*	*g26085.t1*	7	+	5,373,990–5,375,422	325	5.71	37.01	Cytoplasm
*ibGA3ox7*	*g26086.t1*	7	+	5,386,600–5,388,199	364	4.96	40.94	Cytoplasm
*ibGA3ox8*	*g26087.t1*	7	+	5,391,320–5,394,000	387	5.76	43.13	Cytoplasm
*ibGA20ox1*	*g9586.t1*	3	+	1,060,869–1,062,519	358	5.55	40.28	Cytoplasm
*ibGA20ox2*	*g13712.t1*	4	+	6,313,281–6,319,168	454	7.05	51.26	Cytoplasm
*ibGA20ox3*	*g20539.t1*	5	-	28,606,978–28,608,922	387	6.94	43.52	Nucleus
*ibGA20ox4*	*g25334.t1*	7	+	433,867–435,512	360	6.14	40.55	Nucleus

*Chr* Chromosome, * PI* isoelectric point.

Statistical analysis showed that the protein length of most GAox genes was 300–400 aa (89%), with the largest being 532 aa and the smallest being 278 aa. The predicted isoelectric (PI) points had a wide range of 4.94–9.19, and the molecular weights (MWs) of these genes ranged from 30.97 to 59.88 kDa (Table 1 and Supplementary Table S1). The results of the predicted localization of these GAox genes showed that all of the proteins were located in the cytoplasm except for ibGA20ox3 and ibGA20ox4, which was consistent with the properties of GAox genes reported in previous studies^1,3^.

### Multiple sequence alignment and phylogenetic analysis of GAox genes

**Fig. 1:**
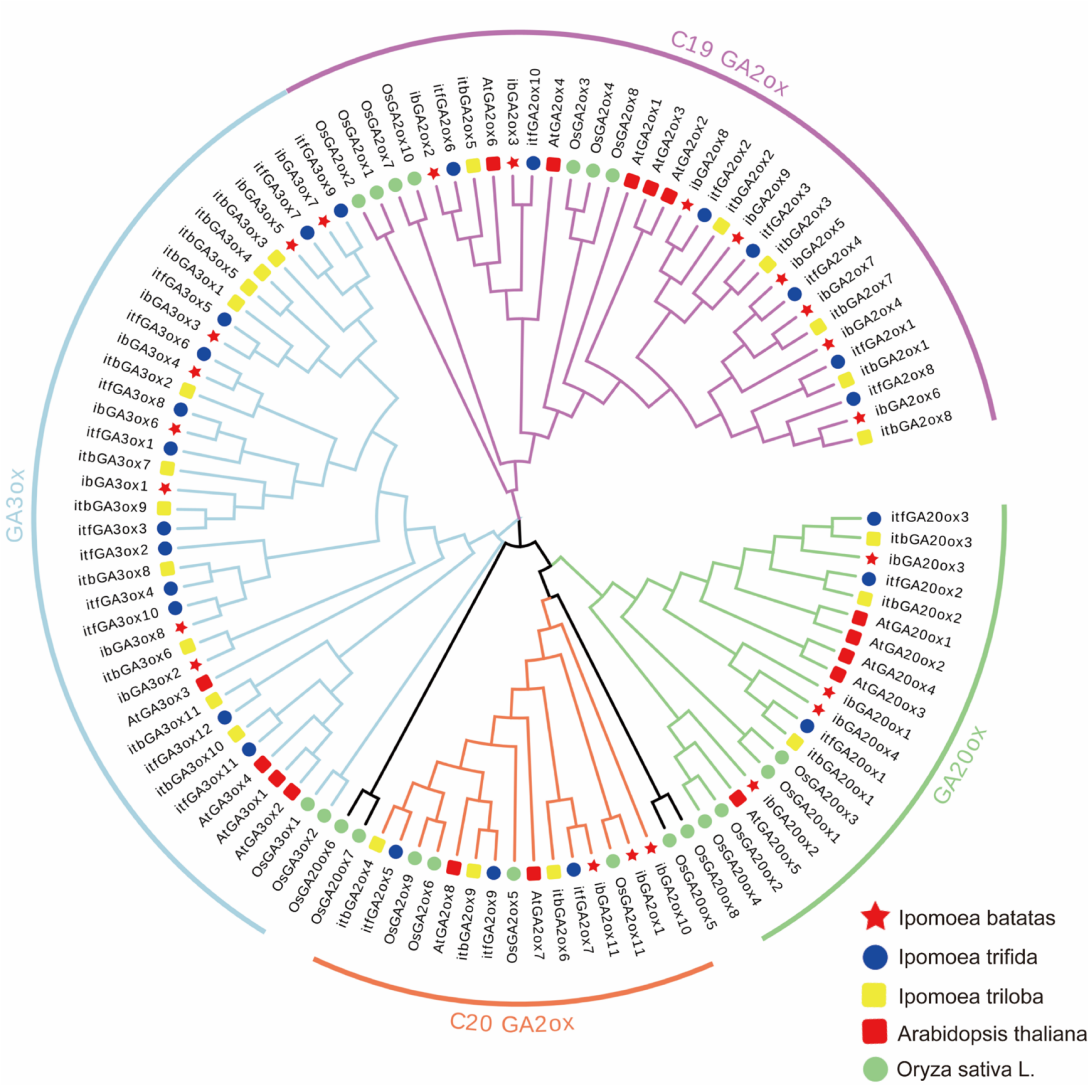
Phylogenetic analysis of gibberellin oxidase in *I. batatas* (ib), *I. trifida* (itf), *I. triloba* (itb), *A. thaliana* (At) and *O. sativa* (Os). A total of 108 GAox except OsGAox6 and OsGAox7 genes were divided into four groups. The red stars, blue circles, yellow squares, red squares, and green circles represent the 23 ibGAoxs in I. batatas, 25 itfGAoxs in I. trifida, 23 itbGAoxs in I. triloba, 16 AtGAoxs in Arabidopsis thaliana and 21 OsGAoxs in Oryza sativa, respectively.

To better investigate the evolutionary relationships and classify the GAox genes, the protein sequences of 16 *AtGAoxs*, 21 *OsGAoxs* and 71 *GAoxs* in the three *Ipomoea* genomes were employed to construct a phylogenetic tree (Fig. 1). The GAox gene family is divided into four groups in *Arabidopsis thaliana* and six groups in rice^8^. The 71 GAox genes identified in sweet potato and its wild relatives were divided into four distinct groups named C19-GA2ox, C20-GA2ox, GA3ox and GA20ox, with the GAox-A, GAox-B and GAox-C groups reported in rice not identified^8^. The results of the phylogenetic analysis were consistent with the previous identification of GAox members, except for four members in rice (Fig. 1). Among the four groups of GAox families in the three *Ipomoea* genomes, GA3ox was the largest clade, containing 31 members, and C20-GA2ox was the smallest, with nine members. GA2ox inhibited the synthesis of bioactive GAs with C19-GAs and C20-GAs as the substrates of C19-GA2ox and C20-GA2ox, respectively^9^. Among the two subgroups of GA2ox, C19-GA2ox contained more members than C20-GA2ox.

### Conserved domain and gene structure analysis of GAox genes

**Fig. 2:**
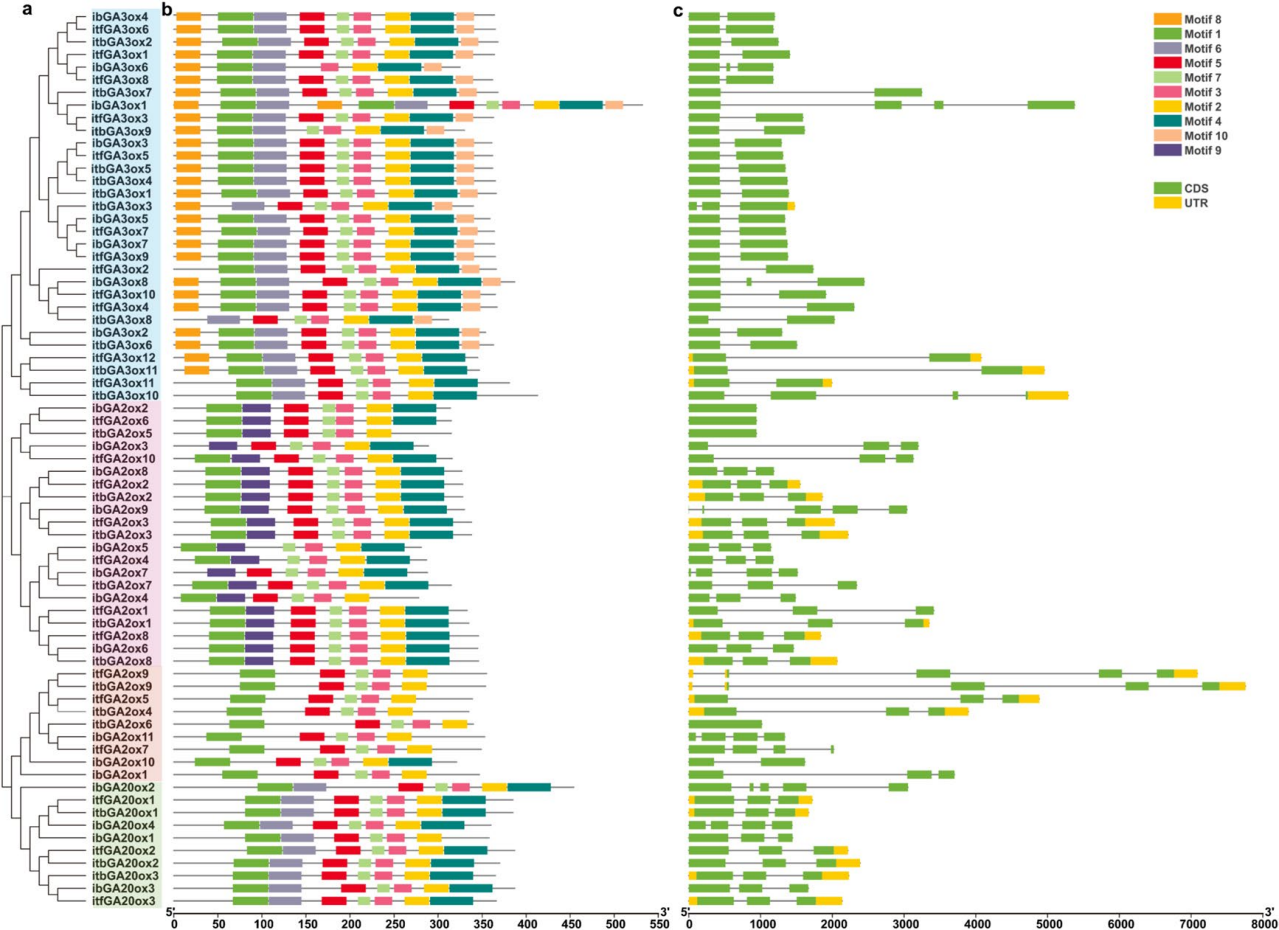
Phylogenetic relationship, gene structure and the conserved protein motifs of GAoxs in *I. batatas*, *I. trifida* and *I. triloba*. (**a**) The phylogenetic tree was constructed based on the protein sequences of GAoxs and they were divided into four subgroups. (**b**) The conserved motif composition. The motifs 1–10 are displayed by different colors. (**c**) Exon–intron structure of GAoxs. Yellow boxes represent UTRs, green boxes represent exons, and the black lines represent introns.

To determine the structural diversity of the GAox family, the conserved domain and gene structure were analyzed and are presented in Fig. 2. The phylogenetic tree of GAox genes constructed for the three *Ipomoea* genomes was consistent with the results described in the previous section related to the four gene groups (Fig. 1 and Fig. 2a). A total of 10 conserved domains among the 71 GAox members were predicted with MEME online analysis (Fig. 2b and Supplementary Table S2). As a whole, the distribution of conserved domains in the members in the GAox family varied between the different subgroups and was relatively similar in the same subgroup (Fig. 2b). In general, the members of GA3ox have 7–9 motifs, except for *ibGA3ox1*, which contains 12 motifs. The consensus motifs among the members of GA3ox genes are motif 2, motif 3, motif 4 and motif 6. The members of C19-GA2ox have 6–7 motifs with the consensus motifs are motif 2, motif 3, motif 7 and motif 9. The subgroup of C20-GA2ox had the fewest motifs (5) compared to the other subgroups in the GAox family. However, *ibGA2ox10* is an exception, with 6 conserved motifs. The number of conserved motifs in most members of the GA20ox genes was 7, with *ibGA20ox* containing 6 conserved motifs. This analysis indicated that the members of the different subgroups of the GAox family contain various conserved domains and therefore perform diverse functions. However, as members of the GAox gene family, they also share some common conserved domains, such as motif 2 and motif 3 *(*Fig. 2b*)*. In summary, the combination of motif 8, motif 1, motif 6, motif 5, motif 7, motif 3, motif 2, motif 4 and motif 10 is unique to the GA3ox subgroup, and the proportion of members of this subgroup with this combination is 67.7%. The typical conserved motif combination of C19-GA2ox is motif 1, motif 9, motif 5 motif 7, motif 3, motif 2, and motif 4, and the proportion of the members of the C19-GA2ox subgroup with this combination is 71.4%. The typical conserved motif combination of C20-GA2ox is motif 1, motif 5 motif 7, motif 3, and motif 2, and the proportion of the members with this combination in the C20-GA2ox subgroup is 88.9%. In the GA20ox subgroup, the conserved motif combination is motif 1, motif 6, motif 5, motif 7, motif 3, motif 2, and motif 4, and the proportion of the members of the GA20ox subgroup with this combination is 90%.

Based on the genomic sequence information, the gene structure of GAoxs is shown in Fig. 2c*.* The intron number of GAoxs varies from 1 to 4. However, *ibGA2ox2*, *itfGA2ox6* and *itbGA2ox5* in the C19-GA2ox subgroup and *itbGA2ox6* in the C20-GA2ox subgroup had no introns (Fig. 2c).

#### Prediction of cis-elements in the promoter region of GAox genes

**Fig. 3:**
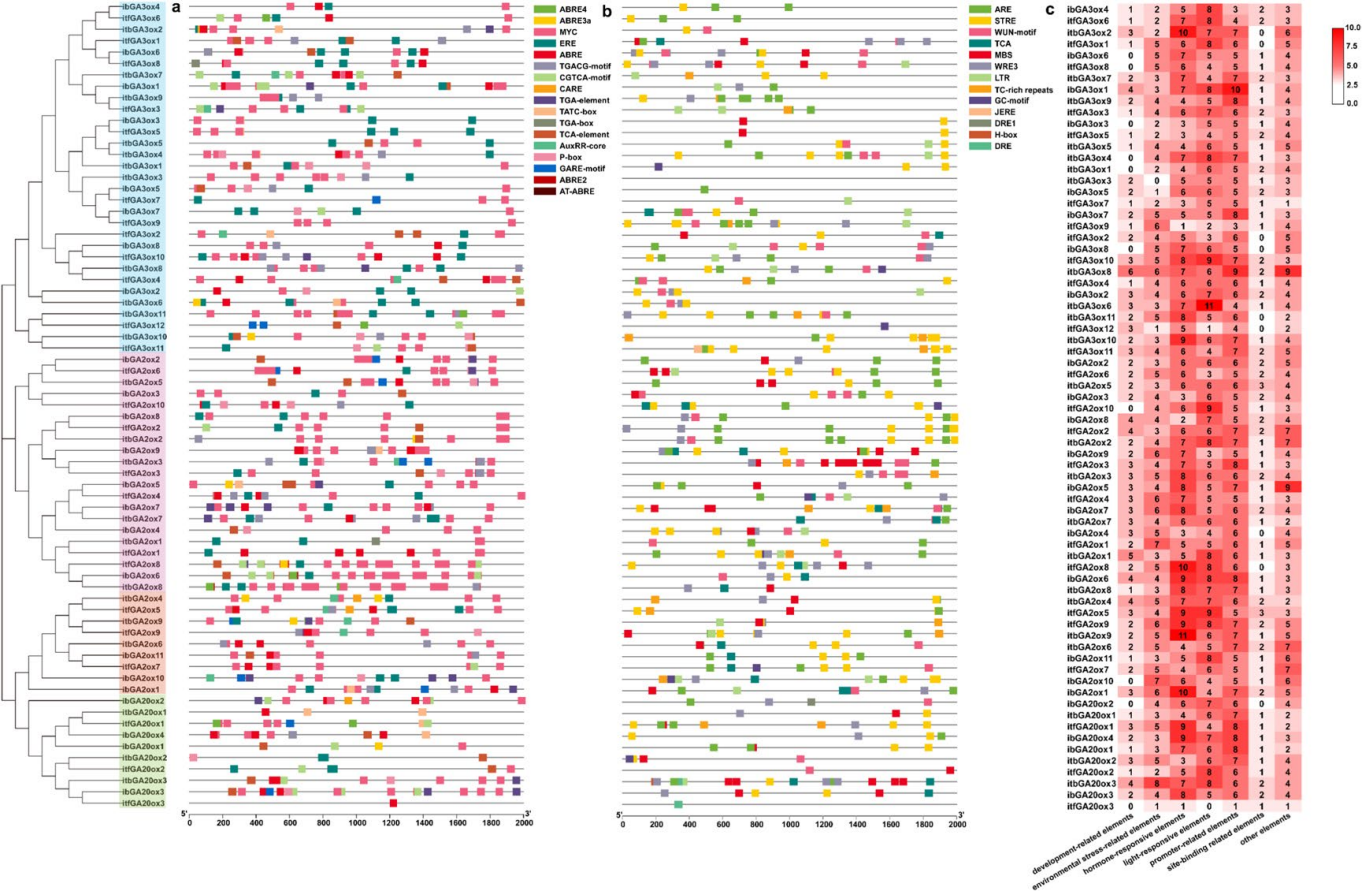
Prediction of *cis*-element in *I. batatas*, *I. trifida* and *I. triloba*. (**a**) The number of *cis*-elements detected in the promoter of GAoxs. All of the *cis*-elements were divided into seven categories. (**b**) Distribution of *cis*-element related to hormone response. (c) Distribution of *cis*-element related to environmental stress in GAoxs.

The 2,000-bp sequence upstream of the start codon in the 71 GAox genes was analyzed to investigate the *cis*-elements in the promoter region (Fig. 3). A total of 106 *cis*-elements were predicted, which were classified into the following seven types: development-related elements, environmental stress-related elements, hormone-responsive elements, light-responsive elements, promoter-related elements, site-binding related elements and other elements (Fig. 3a). The most numerous elements in the promoter region of GAox genes were related to the hormone response, such as the ABRE motif, which is involved in ABA responsiveness; the TGA element, which is related to the IAA response; the MYC motif, involved in methyl jasmonate (MeJA) response; the TATC-box element, related to GA responsiveness; the TCA element, related to salicylic acid (SA) responsiveness; and the ERE motif, which is related to ethylene (ETH) responsiveness. Among these predicted motifs related to hormones, the elements induced by MeJA were the most abundant (Fig. 3b and Supplementary Table S3).

Considering the specific motifs, the most numerous elements were CAAT-box and TATA-box, which are promoter-related elements and were distributed in the promoter region of all GAox genes in the present study except for *ibGA2ox4* (Supplementary Table S3). The next most numerous elements were MYB and Unnamed_4 (68), which belong to the promoter-related and other element types, respectively. The third most numerous elements were MYC and AT-TATA-box (67). MYC is an element involved in MeJA responsiveness, and AT-TATA box is related to the promoter. Although all of the proteins encoded by the three types of genes belong to the GAox family, the promoter regions of 38 genes were predicted to contain GA-related elements, such as *ibGA2ox1*, *ibGA3ox2* and *ibGA20ox2* (Supplementary Table S3). In addition, some elements related to environmental stress were predicted to occur on the promoter regions of these genes, such as the WUN motif, which is a wound-responsive element; the STRE motif, which is activated by heat shock, osmotic stress, low pH, and nutrient starvation; and the LTR motif, which is involved in low-temperature responsiveness (Fig. 3c).

Among these 71 GAox genes, the promoter region of *itfGA2ox5* was predicted to contain the most elements (297 conserved motifs), which covered all seven types of motifs (Supplementary Table S3). However, the promoter region of *itfGA20ox3* contained the fewest conserved motifs (6). The number of motifs in the 23 cultivated sweet potato promoters ranged from 120 to 287.

#### Evolutionary analysis of GAox genes in sweet potato

**Fig. 4:**
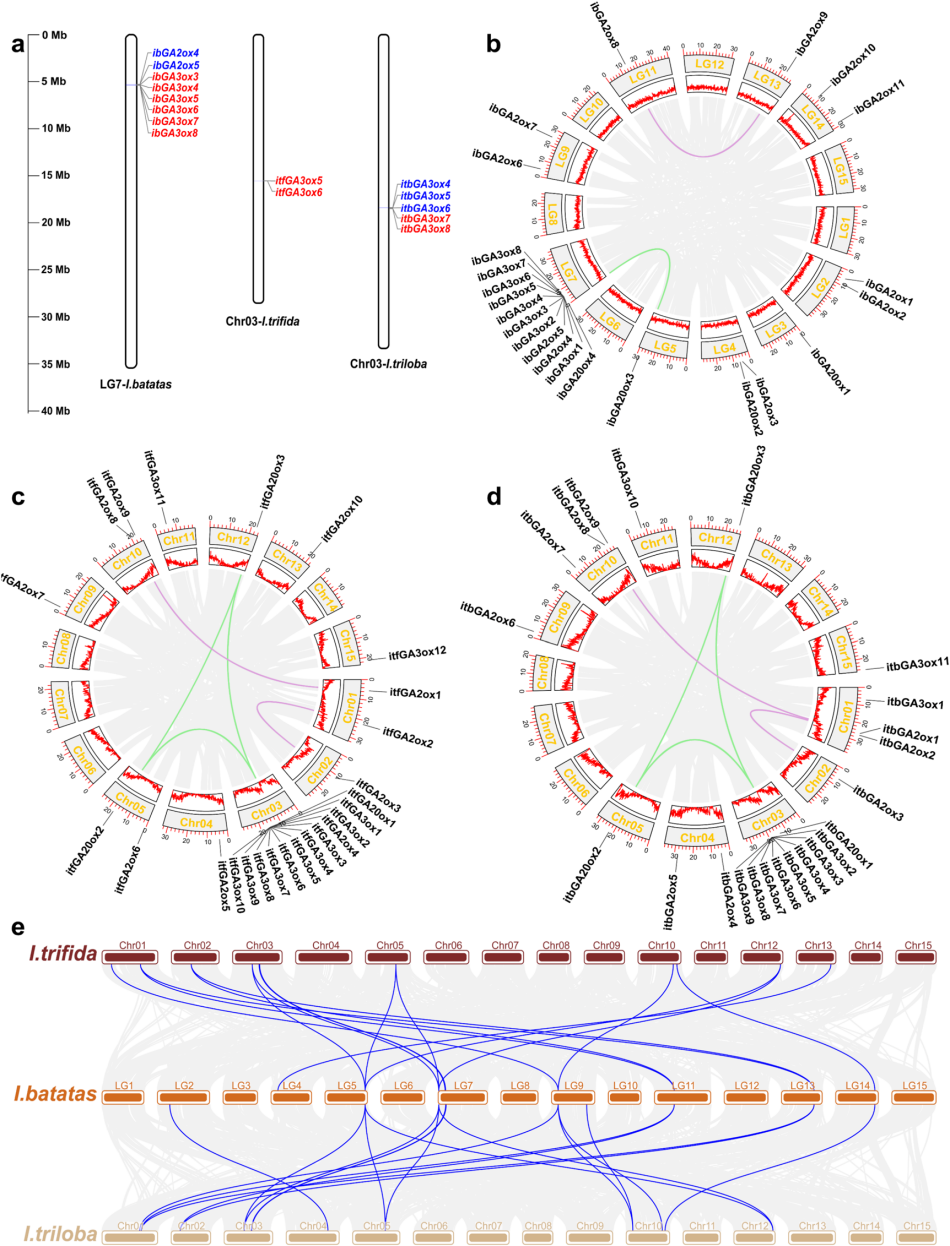
Gene duplication events of the GAox genes occurred in the genomes of *I. batatas*, *I. trifida*, and *I. triloba*, and synteny analyses of the GAox genes were performed between *I. batatas* and its two diploid wild relatives. a Gene duplication events detected in the one chromosome and the different gene duplication events were distinguished by red or blue. b-e Duplication events of GAoxs genes detected in the different chromosomes. The green and purple lines represent the duplication events occurred in GA20ox and GA2ox, respectively. c Synteny analyses of the GAox genes between *I. batatas* and *I. trifida*, and between *I. batatas* and *I. triloba*. Gray lines in the background indicate the collinear blocks in the genome of *I. batatas* with *I. trifida* and *I. triloba*, while the blue lines indicate the syntenic GAox gene pairs.

It has been reported that gene duplication, including tandem duplication and segmental duplication, plays important roles in the expansion of gene families in the genome^21^. Thus, synteny analysis in each of the three *Ipomoea* genomes was conducted to explore the GAox gene expansion states. In the *I. batatas* genome, 6 pairs of GAox genes (*ibGA3ox3*/*ibGA3ox4*, *ibGA3ox4*/*ibGA3ox5*, *ibGA3ox5*/*ibGA3ox6*, *ibGA3ox6*/*ibGA3ox7*, *ibGA3ox7*/*ibGA3ox8*, and *ibGA2ox4*/*ibGA2ox5*) were identified as tandem duplications, and *ibGA3ox3* to *ibGA3ox8* formed a gene cluster in a less than 200-kb genome region (Table 1 and Fig. 4a). A total of two segment duplication events (*ibGA2ox8*/*ibGA2ox9* and *ibGA20ox3*/*ibGA20ox4*) were detected in the various chromosomes (Fig. 4b). In the *I. trifida* genome, only one GAox gene duplication event (*itfGA3ox5*/*itfGA3ox6*) was discovered. Moreover, five pairs of segment duplications (*itfGA2ox1*/*itfGA2ox8*, *itfGA2ox2*/*itfGA2ox3*, *itfGA20ox1*/*itfGA20ox2*, *itfGA20ox1*/*itfGA20ox3*, *itfGA20ox2*/*itfGA20ox3*) occurred in the GAox genes of the *I. trifida* genome (Fig. 4c). Interestingly, a similar situation of segment duplication arose in the *I. triloba* genome (Fig. 4d). A total of three tightly linked GAox genes (*itbGA3ox4*/*itbGA3ox5*, *itbGA3ox5*/*itbGA3ox6*, and *itbGA3ox7*/*itbGA3ox8*) were discovered among the GAox genes of *I. triloba* (Fig. 4a). Although tandem duplication and segment duplication occurred in three *Ipomoea* genomes, the quantity (tandem duplication/segment duplication: 6/2 in *I. batatas*, 1/5 in *I. trifida*, 3/5 in *I. triloba*) of the two types of events was low. Tandem duplication always occurred in the GA3ox subgroup. All these results indicate that tandem duplication might be the primary factor resulting in the expansion of GA3ox genes, and segmental duplication drives the development of GA2ox and C19-GA20ox genes.

The diploid *I. trifida* has been widely regarded as the closest relative of the cultivated sweet potato, and it has reported that *I. batatas* was derived from the alloautopolyploidization of the descendants of *I. trifida* and *I. triloba*^22^. Thus, we analyzed the synteny relationship between the two wild relatives and *I. batatas*. A total of 12 GAox genes in *I. batatas* had collinear relationships with *I. trifida* (16) and *I. triloba* (18) (Fig. 4e). The synteny relationship map shows that some GAox genes in *I. batatas* had a collinear relationship with more than one gene in *I. trifida* or *I. triloba*, such as *ibGA20ox3*/*itfGA20ox1*/*itfGA20ox2*/*itfGA20ox3* and *ibGA20ox3*/*itbGA20ox1*/*itbGA20ox2*/*itbGA20ox3*, which indicated that the ancestor gene of *ibGA20ox3* underwent selection and retained a copy during the evolutionary process. Some other GAox genes occurred in only one pair of collinear genes in *I. trifida* and *I. triloba*, such as *ibGA2ox11*/*itfGA2ox9*/*itbGA2ox9* and *ibGA3ox1*/*itfGA3ox1*/*itbGA3ox2*. The results suggested that a collinear relationship existed prior to the divergence of the two wild relatives.

#### Expression profiles of GAox genes in Ipomoea Genomes

**Fig. 5:**
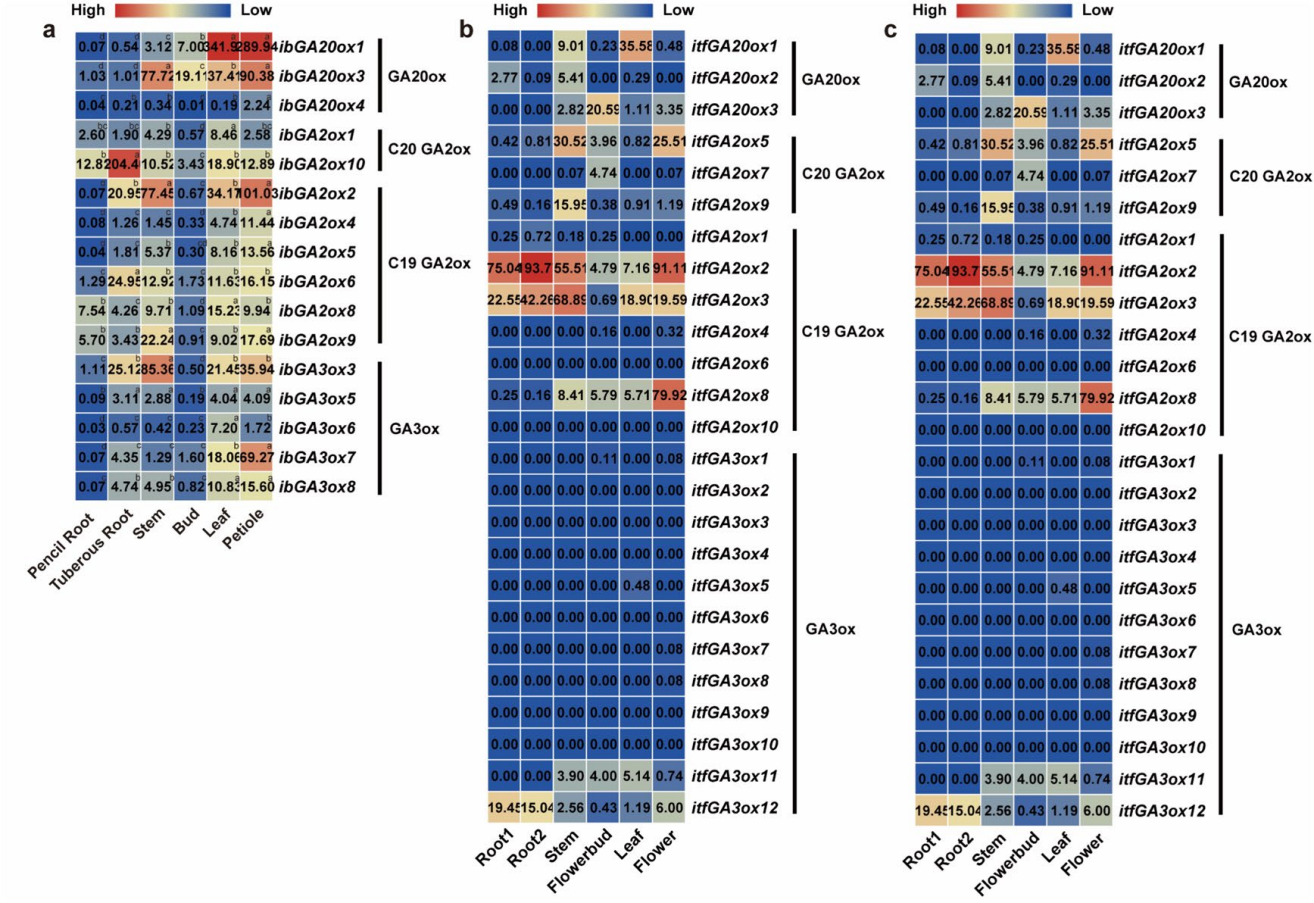
Genes expression pattern of GAox in different tissues of *I. batatas* (**a**), *I. trifida* (**b**) and *I. triloba*(**c**). (**a**) The expression analysis in pencil roots, tuberous root, stem, bud, leaf and petiole of *I. batatas*. The expression level of *ibGA20ox3* in tuberous root was considered as “1”, and the values in the boxes indicate the change fold compared with “1”. Lowercase letters denote statistically significant differences (*p* < 0.05, Student’s *t*-test) among gene expression levels. (**b**) The expression analysis in root1, root2, stem, flower bud, leaf and flower of *I. trifida*. (**c**) The expression analysis in root1, root2, stem, flower bud, leaf and flower of *I. triloba*. Log2(FPKM) from RNA-seq data (http://sweetpotato.uga.edu/) is shown in the box plot.

We determined the expression levels of GAox genes using real-time quantitative PCR (qRT‒PCR) in six representative tissues (i.e., pencil root, tuberous root, stem, bud, leaf, and petiole) to investigate their potential functions in the process of growth and development. Because 16 out of 23 GAox genes were detected to be expressed, subsequent expression analysis was performed on these 16 genes. GAox genes were widely expressed in the six tissues (Fig. 5a). However, different genes showed diverse expression patterns even if the genes were in the same subgroups; for example, *ibGA20ox3* in the GA20ox subgroup was highly expressed in all of the tissues; however, *ibGA20ox4* in the same subgroup showed relatively lower expression (Fig. 5a). Moreover, most of the C19-GA2ox genes showed tissue-specific expression and were highly expressed in petioles and less expressed in buds. Notably, *ibGA2ox8*, *ibGA2ox9* and *ibGA2ox10*, three members of subgroup GA2ox, exhibited relatively higher expression levels in both fibrous roots and tuberous roots. Given the overall gene expression profiles in the sampled tissues, *ibGA2ox10* demonstrates pronounced tissue specificity that corresponds to the reduced gibberellin demand during tuberous root swelling, suggesting its potential role in sweet potato storage root expansion. These results indicated that GAox genes in different parts of cultivated sweet potato play diverse roles during plant growth and development. Moreover, we employed the RNA-seq data of six tissues (root1, root2, stem, flower bud, leaf and flower) to detect the expression pattern of GAox genes in *I. trifida* and *I. triloba*. A total of 8 and 3 GAox genes showed no expression in the selected tissues in *I. trifida* and *I. triloba*, respectively (Fig. 5b and 5c). In *I. trifida*, *itfGA20ox1* and *itfGA20ox3* in the GA20ox subgroup, *itfGA2ox5* and *itfGA2ox9* in the C20-GA2ox subgroup, *itfGA2ox2*, *itfGA2ox3* and *itfGA2ox8* in the C19-GA2ox subgroup, and *itfGA3ox12* in the GA3ox subgroup had higher expression in the different tissues. However, the other GAox genes had lower or rare expression in the five tissues. In addition, a similar situation also occurred in *I. triloba*, for example, *itbGA20ox3* in the GA20ox subgroup, *itbGA2ox4* in the C20-GA2ox subgroup, *itbGA2ox2*, *itbGA2ox3* and *itbGA2ox8* in the C19-GA2ox subgroup, and *itbGA3ox11* in the GA3ox subgroup. These results indicate that in the different subgroups of GAox genes, only some genes play important roles in the growth and development of *I. trifida* and *I. triloba*.

**Fig. 6:**
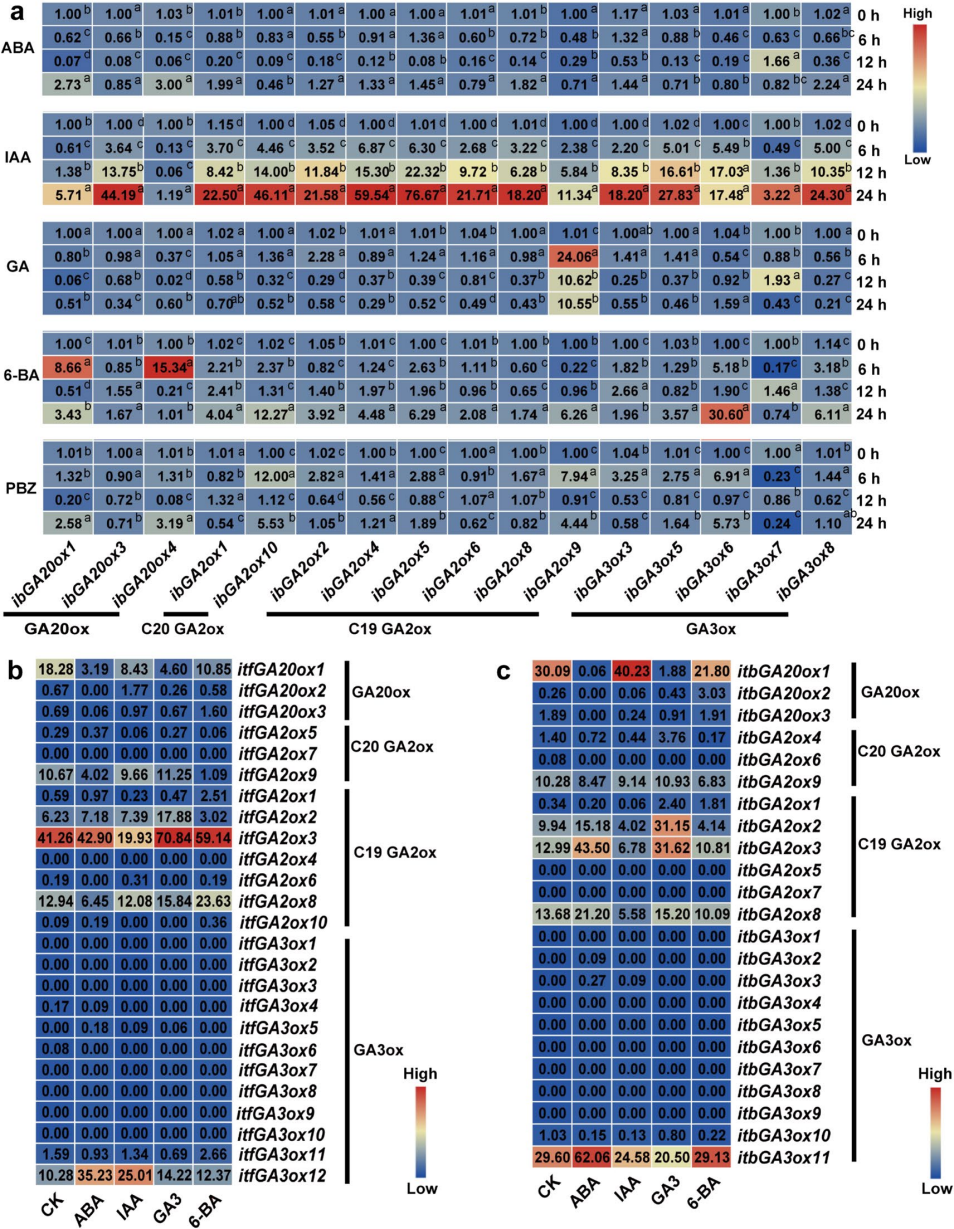
Gene expression patterns of GAoxs in *I. batatas* (**a**), *I. trifida* (**b**) and *I. triloba*(**c**) under the treatment of plant growth regulator. (**a**) The expression of all the GAox gene at the timepoint of 0 h, 6 h, 12 h and 24 h under the treatment of ABA, IAA, GA, 6-benzylaminopurine (6-BA) in *I. batatas*. The expression of 0 h in each treatment was considered as “1”. Lowercase letters denote statistically significant differences (*p* < 0.05, Student’s *t*-test) among gene expression levels. (**b**) The expression analysis under the treatments of control (CK), ABA, IAA, GA3 and 6-BA of *I. trifida*. (**c**) The expression analysis under the treatments of control (CK), ABA, IAA, GA3 and 6-BA of *I. triloba*. Log2(FPKM) from RNA-seq data (http://sweetpotato.uga.edu/) is shown in the box plot and the control (CK) was treated with water in the same manner.

To study the induction of GAox genes by various hormones, we detected the expression levels of GAox genes under treatment with ABA, IAA, GA, 6-benzylaminopurine (6-BA) and PBZ in *I. batatas* (Fig. 6a). Under ABA treatment, *ibGA20ox1* (2.73-fold) and *ibGA20ox4* (3.00-fold) were significantly induced at 24 h after treatment. However, *ibGA2ox10* was significantly repressed at 12 h and 24 h after treatments. Under IAA treatment, all of the GAox genes were significantly induced at 24 h after treatment, especially *ibGA2ox4* (59.54-fold) and *ibGA2ox5* (76.67-fold). Under GA treatment, *ibGA2ox2*, *ibGA2ox5*, *ibGA2ox6* and *ibGA2ox9* in the subgroup of GA2ox was significantly induced. In addition, all the members of the subgroup of GA20ox and *ibGA3ox8* in the GA3ox subgroup were significantly repressed. Under 6-BA treatment, all the members of GAox genes in *I. batatas* were induced, especially *ibGA3ox6* (30.60-fold), at 24 h after treatment. *ibGA20ox1* and *ibGA20ox4*, members of the GA20ox subgroup, exhibited significantly high expression levels under PBZ treatment at 24 h after treatment. Meanwhile, in the GA3ox subgroup, *ibGA3ox3*, *ibGA3ox5*, *ibGA3ox6*, *ibGA3ox7* and *ibGA3ox8* displayed the same trend following *ibGA20ox1* and *ibGA20ox4*. However, *ibGA2ox1* and *ibGA2ox6* in the GA2ox subgroup showed a completely opposite expression pattern (Fig. 6a).The above results indicate that most of the GAox genes respond to hormone treatment to varying degrees. We also investigated the expression levels of GAox genes under treatment with different plant hormones in *I. trifida* and *I. triloba* (Fig. 6b and Fig. 6c). A total of 16 GAox genes showed expression in *I. trifida*. The genes *itfGA20ox1*, *itfGA3ox4* and *itfGA3ox6* were repressed by all four plant hormones. In contrast, *itfGA3ox12* was highly induced by the four plant hormones. In *I. triloba*, *itbGA2ox6* and *itbGA3ox10* were repressed by all four plant hormones.

**Fig. 7:**
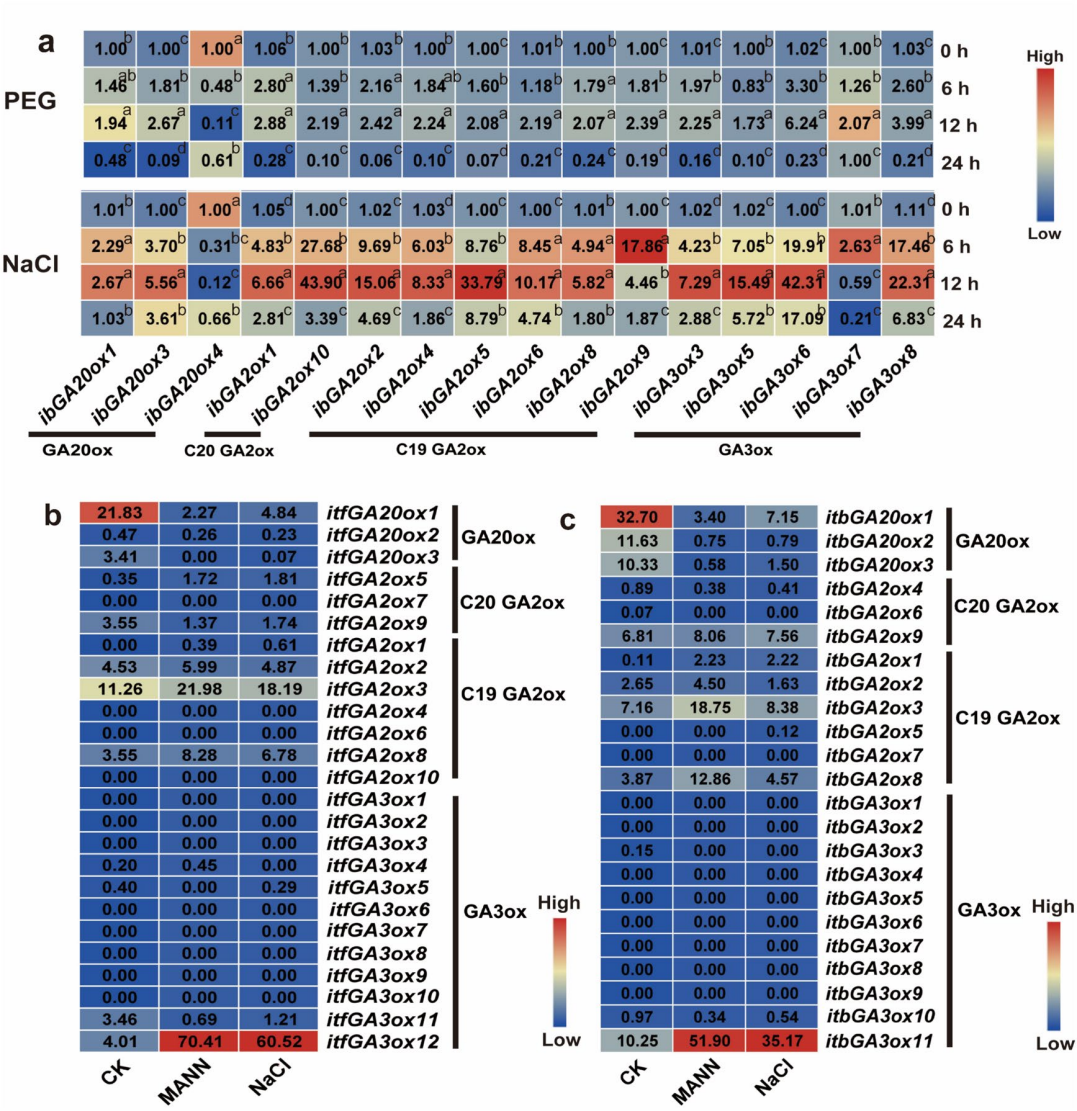
Gene expression patterns of GAoxs in *I. batatas* (**a**), *I. trifida* (**b**) and *I. triloba*(c) under the treatment of simulated soil drought treatment (PEG) and NaCl. (**a**) The expression of all the GAox gene under the treatment of PEG and NaCl in *I. batatas*. The expression of 0 h in each treatment was considered as “1”. Lowercase letters denote statistically significant differences (*p* < 0.05, Student’s *t*-test) among gene expression levels. (**b**) The expression analysis under the treatments of control (CK), mannitol (MANN) and NaCl of *I. trifida*. (**c**) The expression analysis under the treatments of control (CK), control (CK), mannitol (MANN) and NaCl of *I. triloba*. Log2(FPKM) from RNA-seq data (http://sweetpotato.uga.edu/) is shown in the box plot and the control (CK) was treated with water in the same manner.

To investigate whether GAox genes in *I. batatas* are involved in abiotic stress response, we evaluated the expression levels of these genes under PEG and NaCl treatment (Fig. 7a). All of the GAox genes were significantly induced by PEG except *ibGA20ox4*. In addition, most of these genes, except *ibGA20ox4*, were also significantly induced by NaCl, especially at 12 h after treatment. Based on the RNA-seq data of *I. trifida* and *I. triloba* treated with mannitol and NaCl (Fig. 7b and Fig. 7c), all the members of the GA20ox subgroup were repressed in the two wild relatives of *I. batatas*. In addition, *itfGA2ox9*, *itfGA3ox5* and *itfGA3ox11* in *I. trifida* and *itbGA2ox4*, *itbGA2ox6*, *itbGA3ox3* and *itbGA3ox10* in *I. triloba* were also repressed. However, some genes were highly induced by mannitol and NaCl, such as *itfGA3ox12* and *itbGA3ox11* (Fig. 7b and Fig. 7c). Taken together, these results indicate that GAox genes might participate in the abiotic stress response.

### Co-expression analysis of GAox genes in response to hormones and abiotic stress in sweet potato

**Fig. 8:**
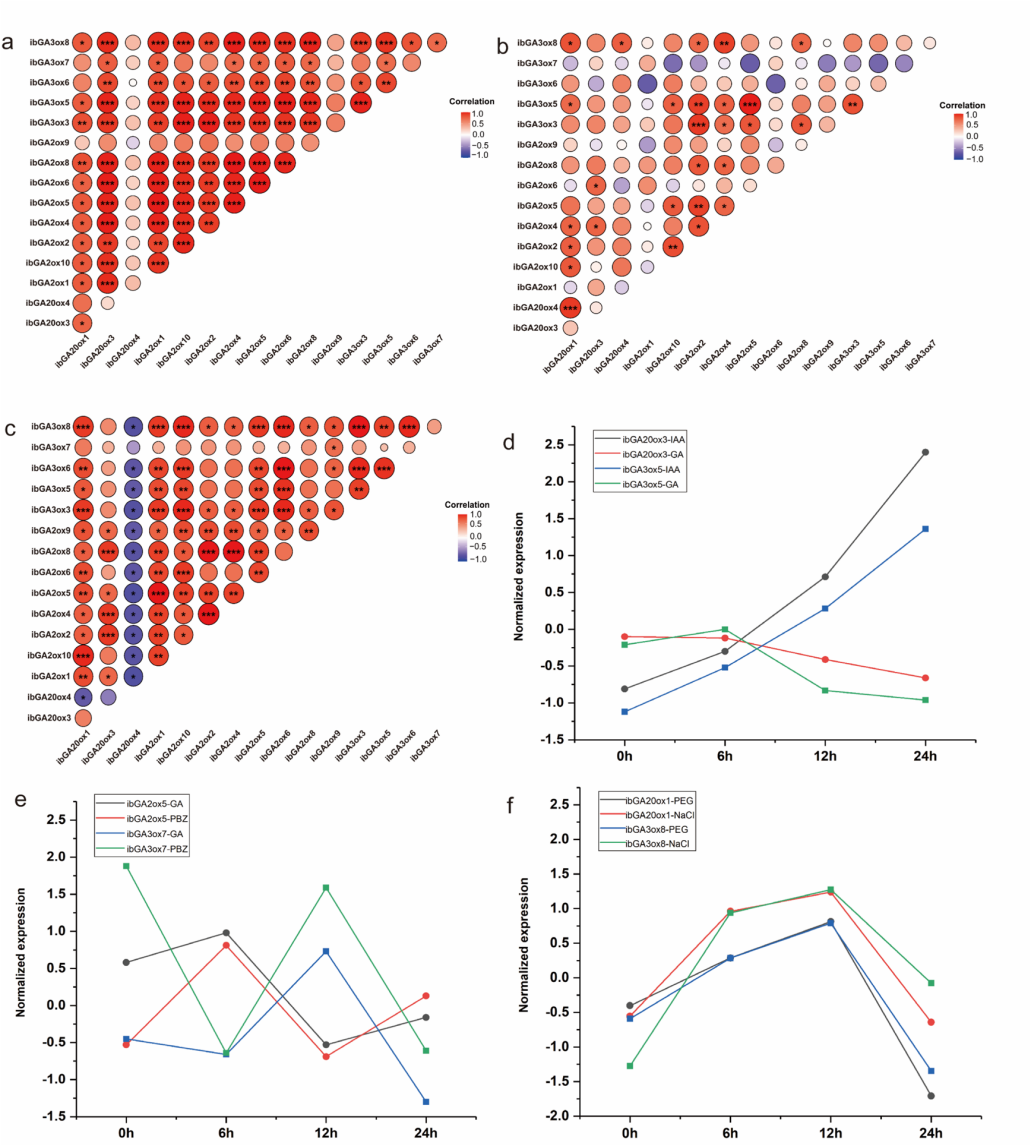
Correlations of GAox genes and expression patterns of *IbGA20ox1*, *IbGA20ox3*, *IbGA2ox5*, *IbGA3ox5*, *IbGA3ox7* and *IbGA3ox8* under hormones and abiotic stress in sweet potato. (**a**, **b**, **c**) Correlation analysis using the R package program. Each correlation is shown by the shades of blue and red and the size of the circle shape. *, ** and **represent correlations with *P* value ≤ 0.05, *P* value ≤ 0.01 and *P* value ≤ 0.001 respectively. (**d**) expression patterns of *IbGA20ox3* and *IbGA3ox5* under IAA and GA treatments. (**e**) expression patterns of *IbGA2ox5* and *IbGA3ox7* under GA and paclobutrazol (PBZ) treatments. (f) expression patterns of *IbGA20ox1* and *IbGA3ox8* under NaCl and PEG treatments. Expression data were standardized using the Z-score method.

Given the distinct hormonal responses of sweet potato seedlings, this study focused on the co-expression networks of gibberellin oxidase (GAox) genes under treatments with gibberellin (GA), auxin (IAA), paclobutrazol (PBZ), and abiotic stresses (Fig. 8). Under IAA and GA treatments, most GAox genes exhibited strong positive correlations. For instance, *ibGA20ox3* was closely correlated with *ibGA2ox4* (r = 0.990, *P* < 0.001), and *ibGA2ox4* with *ibGA2ox8* (r = 0.997, *P* < 0.001). Notably, *ibGA20ox3* and *ibGA3ox5* functioned as hub genes, showing synchronized expression with multiple GA2ox members (Fig. 8a). Between GA biosynthetic genes (GA20ox, GA3ox) and inactivation genes (GA2ox), widespread co-expression was observed. Under GA and PBZ treatments, significant correlations were identified across pathways, such as *ibGA2ox5* with *ibGA3ox5* (r = 0.99, *P* < 0.0001) and *ibGA2ox2* with *ibGA3ox3* (r = 0.93, *P* < 0.001), indicating coordinated regulation rather than simple antagonism. Although less frequent, antagonistic relationships were also detected, exemplified by *ibGA3ox7* showing negative correlations with *ibGA2ox5* and *ibGA3ox5* (Fig. 8b). Under NaCl and PEG stresses, gibberellin oxidase genes exhibited highly coordinated expression patterns. Specifically, *ibGA20ox1* and *ibGA3ox8* showed a significant positive correlation (r = 0.929, *P* < 0.001), indicating their synergistic regulation in the GA biosynthetic pathway under stress conditions. In contrast, *ibGA20ox4* demonstrated significant negative correlations with most genes, including antagonistic relationships with *ibGA20ox1* (r = -0.714) and *ibGA3ox8* (r = -0.786), revealing opposing regulatory modules within the GA metabolic network (Fig. 8c). These findings suggest that the maintenance of GA homeostasis under stress conditions relies on both coordinated expression among biosynthetic genes and antagonistic regulation by specific genes.

Expression analysis revealed distinct regulatory patterns among GA metabolic genes: *ibGA20ox3* responded rapidly to IAA, whereas *ibGA3ox5* exhibited a sustained response to GA (Fig. 8d). Under paclobutrazol (PBZ) treatment, *ibGA2ox5* was suppressed, while *ibGA3ox7* was induced, indicating compensatory regulation within the GA pathway (Fig. 8e). Under NaCl and PEG-induced stresses, the biosynthetic genes *ibGA20ox1* and *ibGA3ox8* were transiently upregulated at 6 h but declined below basal levels by 24 h, revealing a biphasic expression pattern consistent with early stress activation followed by suppression (Fig. 8f). These findings illustrate how GA metabolism is dynamically balanced through coordinated synthesis and degradation to maintain hormonal homeostasis under varying environmental and hormonal conditions.

#### Subcellular localization of GAox proteins in *I. batatas*

**Fig. 9:**
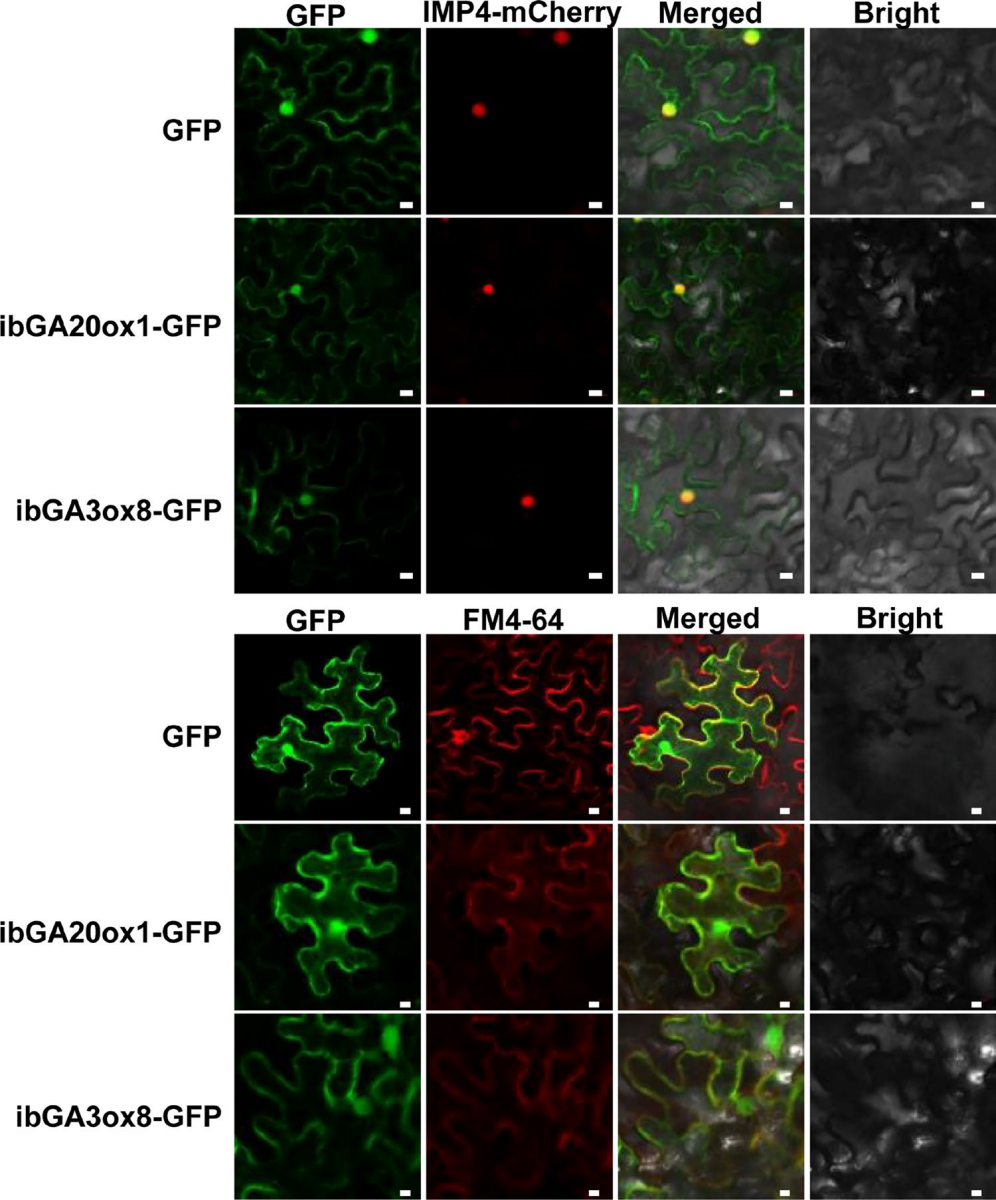
Subcellular localization of ibGA20ox1 and ibGA3ox8 proteins. The pFGC5941::GFP (GFP) vector was used as a control. IMP4-mCherry served as the nuclear marker and FM4-64-mCherry served as the plasma membrane marker. Bar, 10 μm.

We picked two GAox genes in *I. batatas* genome (*ibGA20ox1* and *ibGA3ox8*) to detect their locations of corresponding proteins (Fig. 9). The coding sequences of *ibGA20ox1* and *ibGA3ox8* without stop codons from Shangshu19 were cloned and inserted into the modified pFGC5941 vector that carry the the green fluorescent protein (GFP) gene. Then the fusion expression vector was transformed into the *N. benthamiana* leaves via Agrobacterium tumefaciens strain EHA105. The results showed that the GFP fluorescence signal of *ibGA20ox1* and *ibGA3ox8* were widely present in the cell membrane, nucleus and cytoplasm (Fig. 9).

## Discussion

Gibberellins are important phytohormones associated with diverse aspects of growth and development, as well as resistance to biotic or abiotic stresses^23^. The amount of active GAs is crucial for plants to maintain normal growth and development in changeable circumstances. As the fourth most significant crop in China, sweet potato was once considered a life-saving food. Although the function of GAox genes has been clarified in other crops^13,16,24,25^, their regulatory mechanisms remain largely unclear in sweet potato. In this study, we identified GAox genes in the genomes of sweet potato and its diploid wild relatives, and analyzed their characteristics and expression patterns in different organs and under the treatment of different phytohormones and abiotic stresses to gain a better understanding of GAox gene function in sweet potato.

### Identification, gene structure and phylogenetic relationship analysis

In this study, a total of 71 GAox genes were identified in the genomes of *I. batatas*, *I. trifida* and *I. triloba*. The number of GAoxs identified in *I. batatas* (23) was the same as that in *I. triloba* (23), with two more identified in *I. trifida* (25) than in *I. batatas* (Table 1). Despite its larger genome, the hexaploid sweet potato shows no expansion in GAox gene number compared to its diploid relatives. This contrasts with typical polyploids like wheat (63)^26^ and sea-island cotton (428)^27^, where gene numbers greatly exceed those in diploids such as rice (21) and *Arabidopsis* (16)^9^. Such expansions enable functional specialization; in cotton, *Gh_A13G1787* is expressed specifically in stems, while *Gh_D09G0746* is active only in leaves^27^. The lack of GAox gene expansion in sweet potato indicates strong diploidization and gene loss after polyploidization, maintaining only a core set of genes. This reveals the divergence in evolutionary trajectories of gene families following polyploidization across different crops.

The GAox genes included the GA3ox, GA20ox and GA2ox genes. GA20ox and GA3ox catalyze the formation of bioactive GAs, while bioactive GAs and their immediate precursors are inactivated by GA2ox genes^28^. Based on a previous study, GA2ox can be divided into two subgroups: C19-GA2ox and C20-GA2ox^7,29^. In the current study, the GAox genes were divided into four subgroups based on the results of the phylogenetic tree, which was consistent with previous studies (Fig. 1). However, *OsGA20ox5*, *OsGA20ox6*, *OsGA20ox7* and *OsGA20ox8* were not classified into the GA20ox subgroup in our results, similarly to other studies^29^. Further experiments are needed to confirm whether these genes belong to the GA20ox subgroup. The four types of GAox genes all belong to the 2-ODD superfamily and share some common conserved domains^30^. Due to the completely different functions of the genes in the four subgroups, some unique motifs occurred only in specific subgroups. In our results, motif 6 is specifically contained in the subgroups catalyzing the formation of bioactive GAs (GA3ox and GA20ox) compared with the subgroups catalyzing bioactive GA degradation. Moreover, motif 8 and motif 10 can be used to distinguish GA3ox from GA20ox. The different motifs between C19-GA2ox and C20-GA2ox are motif 9 and motif 4, which occurred only in C19-GA2ox. Motif 1, motif 5, motif 7, motif 3 and motif 2 are the common motifs shared by all the GAox genes. From our results, the four subgroups can be distinguished from each other through motifs while maintaining commonality, which may be due to functional differentiation of the 2-ODD superfamily during evolution. Further experiments are needed to determine the specific functions of these motifs and whether these motifs are specifically related to substrate binding.

### Functions of GAox genes during the swelling of sweet potato storage roots and related to hormone response in sweet potato

The GA2ox subfamily serves as a core regulatory component in storage root development, promoting root expansion and starch accumulation through the reduction of bioactive gibberellin levels. This conserved mechanism is clearly exemplified in potato, where *StGA2ox1* facilitates tuber initiation by lowering bioactive GA, while its suppression leads to developmental delays and aberrant tuber morphology^31^. Tissue-specific expression analysis demonstrated that *ibGA2ox10* is predominantly expressed in storage roots, exhibiting a transcript level of 204.40—the highest among all tissues examined. This pronounced tissue specificity underscores its potential functional significance in storage root development. As a member of the GA2ox subfamily, *ibGA2ox10* catalyzes the conversion of bioactive GAs into inactive forms. This activity is critical for establishing a "low-GA environment," a key physiological trigger that restrains longitudinal cell elongation, promotes starch accumulation, and consequently drives radial swelling—the hallmark of storage root expansion. The expression level of *ibGA2ox10* in storage roots (204.40) was 15.9-fold greater than that in fibrous roots (12.81), indicating substantial transcriptional activation during the transition from fibrous to storage roots. This pronounced upregulation strongly nominates *ibGA2ox10* as a key candidate gene regulating storage root swelling. By contrast, other genes with relatively high expression in storage roots—such as *ibGA3ox3* (25.12), *ibGA2ox2* (20.95), and *ibGA2ox6* (24.95)—either maintain considerable expression in other tissues or display markedly lower transcript levels in storage roots relative to *ibGA2ox10*.

The results of promoter *cis*-element analysis showed that GAox genes contained different types of elements related to hormone responses, such as ABRE, TAG, MYC, TATA-box, TCA-motif and ERE-motif (Fig. 3). Complicated crosstalk has been reported between GA and other plant hormones. For example, GA interacts with auxin to control the elongation of Arabidopsis hypocotyls^32^. The expression analysis showed that most of the GAox genes identified in this study were responsive to the induction of different plant hormones at varied levels, which was consistent with the diverse *cis*-elements related to plant hormones in the promoter region of GAox genes. Under GA and PBZ treatments, a significant positive correlation was observed between certain synthase genes (e.g., *ibGA3ox5*) and catabolic enzyme genes (e.g., *ibGA2ox5*) (Fig. 8b). This phenomenon diverges from the traditional antagonistic model and may reflect a coordinated regulatory mechanism for maintaining GA homeostasis in plants. When bioactive GA levels rise, plants may simultaneously activate both biosynthetic and degradative pathways to achieve precise hormonal control through rapid metabolic turnover. *ibGA3ox7* exhibited a negative correlation with several other members, particularly with *ibGA2ox5* (r = -0.69, *p* = 0.056). Although not highly significant, this trend suggests a potential antagonistic relationship, possibly reflecting a dynamic balance between GA synthesis and degradation pathways. Under GA treatment, the expression patterns of *ibGA3ox7* and *ibGA2ox5* largely aligned with the classical negative feedback regulation model: elevated GA levels suppress synthesis and enhance degradation to restore homeostasis. However, under paclobutrazol (PBZ) treatment, these functionally antagonistic genes exhibited responses contrary to the canonical model: under GA-deficient stress, the synthase gene *ibGA3ox7* was not strongly induced as expected, while the catabolic gene *ibGA2ox5* was upregulated during later stages (Fig. 8e). This indicates that under chemical stress, the regulation of GA metabolism in plants may be governed by a more complex mechanism that extends beyond simple homeostatic feedback.

### Functions of GAox genes related to abiotic stress response in sweet potato and its wild relatives

Previous studies have reported that GAox genes participate in diverse abiotic stress responses in plants^33^. Low GA activity promotes drought avoidance responses, and water deficiency inhibited the expression of the GA biosynthesis genes *GA20ox1* and *GA20ox2* and induced the GA deactivating gene *GA2ox7* in guard cells and leaf tissue in tomato^34^. The same situation also occurs in rice, and water deficiency inhibits GA biosynthesis, with the expression of *GA20ox1* and *GA3ox2* being repressed and that of *GA2ox3,7,8,9* being induced^35^. The GA signaling pathway has also been reported to be involved in the response to NaCl stress. It has been reported that the application of GA3 significantly promoted plant height and fresh/dry weight but was markedly hindered under NaCl treatment^36^. In upland cotton, overexpression of *GhGA2ox1* resulted in higher salt tolerance than in nontransgenic cotton plants^37^. Our study revealed that under PEG and NaCl treatments, most GAox genes in the gibberellin (GA) biosynthesis pathway exhibited positive correlations, with the expression levels of *GA20ox1* and *GA3ox8* showing a highly significant positive correlation (Fig. 8c). Their expression was synchronously upregulated during the early stress stages (6 h and 12 h) but dropped sharply at 24 h (Fig. 8f). This temporal expression pattern demonstrates that plants do not immediately inhibit growth upon stress exposure. Instead, the GA metabolic pathway undergoes a dynamic adaptation process: it is transiently activated initially to potentially promote growth for stress escape or defense priming, and then switches to active growth inhibition to conserve resources for survival when the stress persists, thereby facilitating a fundamental strategic shift from “growth” to “defense”.

In summary, we preliminarily explored the function of GAox genes in sweet potato and its two wild relatives based on the expression analysis results in different organs, treatment with different plant hormones and abiotic stresses and provided valuable information for GA signaling pathway research in sweet potato. However, further experiments are needed for more accurate functional investigation.

## Materials and methods

### Characterization of the GA2ox, GA3ox and GA20ox genes in the genomes of Ipomoea

The sequences of *Arabidopsis thaliana* and *Oryza sativa* were downloaded from Phytozome (https://phytozome-next.jgi.doe.gov/). The sweet potato cultivar sequence cv. Taizhong 6 was obtained from the sweet potato genome database (http://ipomoea-genome.org), and the sequences of *I. trifida* and *I. triloba* were obtained from the sweet potato genomics resource database (http://sweetpotato.uga.edu/). BLAST was performed in three *Ipomoea* genomes based on the GAox family gene sequences in Arabidopsis. Then, we downloaded the hidden Markov model (HMM) file of GAox gene domains (PF14226 and PF03171) from the Pfam database (http://pfam.xfam.org). The GAox protein sequences were obtained using HMMER 3.0 with a cutoff of 0.01 from the three Ipomoea genomes^38^. The high-confidence and nonredundant genes were identified as members of the GAox family. Then, the candidate GAox genes in the three *Ipomoea* genomes were named according to their homology in *Arabidopsis thaliana*. Ultimately, the basic characteristics of proteins encoded by the genes of the GAox family were obtained by ExPASy (http://web.ExPASy.org/protparam/), and the subcellular localization was predicted by Cell-PLoc 2.0 (http://www.csbio.sjtu.edu.cn/bioinf/Cell-PLoc-2/).

### Chromosomal distribution and duplication of GAox genes

The chromosomal distribution of GAox genes in *I. batatas* was labeled based on its GFF3 files and visualized by MG2C online software (http://mg2c.iask.in/mg2c_v2.1/). The duplication events of the GAox gene in *I. batatas* were analyzed by the Multicollinearity ScanToolkit (MCScanX) with the default parameters^39^. TBtools software was used to visualize the collinear relationship between the GAox genes of the three *Ipomoea* genomes^40^.

### Phylogenetic, conserved motif, gene structure and promoter cis-element analysis

Based on the protein sequences of GAox in *Arabidopsis thaliana*, *Oryza sativa*, *I. batatas*, *I. trifida* and *I. triloba*, MEGA7 was employed to construct the phylogenetic tree with the neighbor-joining method and 1,000 bootstrap replications^41^. The conserved motifs were analyzed by the online program Multiple EM for Motif Elicitation (https://meme-suite.org/index.html) in the three Ipomoea genomes^42^. The number of motifs and motif width ranges were set as 20 and 6 to 50 residues, respectively. The gene structure was obtained with the GFF3 files of the three Ipomoea genomes. The conserved motif and gene structure were visualized by TBtools software^40^.

### Promoter cis-element analyses of GAox genes in the three Ipomoea genomes

The 2,000-bp sequence upstream of the start codon was used to analyze the promoter *cis*-elements. The *cis*-elements in the promoter regions of GAox genes were analyzed by PlantCARE^43^.

### Expression analysis of GAox genes in the three Ipomoea genomes

The RNA-seq data of *I. trifida* and *I. triloba* in various tissues and under different types of biotic and abiotic stresses were taken from the resources provided by the sweet potato genome database (http://sweetpotato.uga.edu/). The expression level of GAox genes was represented by fragments per kilobase million (FPKM) values and Log2 (FPKM) was shown in the boxes and visualized in the form of a heatmap. The sweet potato (*I. batatas*) cultivar Shangshu19 was used for expression analysis. Shangshu19 was planted in the campus field of Henan University of Science and Technology, Luoyang, China with in a row 2 m in length, 0.90 m in width, and 0.20 m in interplant spacing. Total RNA was extracted from six tissues (buds, petioles, stems, leaves, pencil roots and tuberous roots) of *I. batatas* for analyzing the tissue-specific expression of GAox genes, with three biological replicates included for each tissue. For the expression analysis of GAox genes after treatment with a growth regulator and abiotic stress, vigorous Shangshu19 seedlings with essentially uniform growth were chosen, and apical 4–5 stem nodes were excised for sand-based pot cultivation. The 4th or 5th expanded leaves on the main stem of Shangshu19 were sampled at 0 h, 6 h, 12 h and 24 h after being sprayed with 100 µM ABA, 100 µM 6-BA, 100 µM GA, 100 µM (indole-3-acetic acid) IAA and 300 µM PBZ, respectively. To detect the response of GAox genes to abiotic stress, the 4th or 5th expanded leaves on the main stem of Shangshu19 were sampled at 0 h, 6 h, 12 h and 24 h after the plants were treated with 1/2 strength Hoagland’s solution with 30% PEG 6000 and 200 mM NaCl. For each treatment, one leaf was sampled per pot of sweet potato, with three pots designated as three biological replicates. Total RNA was extracted with the TRIzol method (Vazyme, China), and the first-strand cDNA template was obtained using a cDNA synthesis kit (R222-01; Vazyme, China). qRT‒PCR was conducted with the SYBR detection protocol (Q311-02/03; Vazyme, China) on an ABI 7500 system (Applied Biosystems, USA). The sweet potato actin gene (GenBank number: AY905538) was selected as an internal control^44^. The relative expression was quantified as the 2^-ΔΔCT^ value^45^. The sequences of primers used in this study are listed in Supplemental Table S4.

### Pearson correlation and expression pattern analysis

Pearson correlation analysis of qRT-PCR results was performed using R package, and a correlation matrix was plotted. To mitigate technical variations, gene expression values (GEV) were normalized by log₂ (GEV + 1) transformation, followed by Z-score scaling across rows for heatmap visualization^46^.

### Subcellular localization

The full-length CDSs of *ibGA20ox1* and *ibGA3ox8* without stop codons were amplified by PCR with specific primers (Supplementary Table S5) from the cDNA of sweet potato cultivar Shangshu19. The obtained fragments were inserted into the modified vector of *pFGC5941*, which carried the GFP reporter gene. Then, the fusion expression vectors and the control vector 35S:GFP were transformed into *A. tumefaciens* strain EHA105 and permeated into *N. benthamiana* leaves. Fluorescent signals were monitored using a confocal laser scanning microscope (LSM710; Zeiss). The GFP was excited at 488-nm excitation and 110-nm collection bandwidth.

## Supplementary Information

Supplementary Information 1.

## Data Availability

All relevant data are within the manuscript and its Supplementary Materials.
