# Supplementary material for: Genome wide identification and expression analysis of gibberellin oxidase family genes in sweet potato and its two diploid relatives
Source: Sci Rep. 2026 Feb 1;16:6882. doi: 10.1038/s41598-026-37951-8 (PMC12916950; doi:10.1038/s41598-026-37951-8)
Supplement: Supplementary file 1 — Supplementary Information 1. [file 41598_2026_37951_MOESM1_ESM.zip › Supplementary materials/Table S1.docx]

**Table S1 Putative GAox genes in *I. trifida* and *I. triloba*.**

| **Gene symbol** | **Gene ID** | **Chr** | **Strand** | **Position** | **Peptide length (aa)** | **PI** | **MW (kDa)** | **Predicted localization** |
| --- | --- | --- | --- | --- | --- | --- | --- | --- |
| *itfGA2ox1* | *itf01g07060.t1* | 1 | - | 5410042-5413457 | 332 | 6.20 | 37.10 | Cytoplasm |
| *itfGA2ox2* | *itf01g20990.t1* | 1 | + | 22005741-22007293 | 327 | 7.67 | 36.31 | Cytoplasm |
| *itfGA2ox3* | *itf02g14170.t1* | 2 | - | 11390076-11392108 | 337 | 6.56 | 37.55 | Cytoplasm |
| *itfGA2ox4* | *itf03g19700.t1* | 3 | - | 15480692-15481871 | 286 | 6.25 | 31.96 | Cytoplasm |
| *itfGA2ox5* | *itf04g06990.t1* | 4 | - | 4216414-4221299 | 338 | 7.19 | 37.99 | Cytoplasm |
| *itfGA2ox6* | *itf05g00500.t1* | 5 | + | 506653-507597 | 314 | 9.19 | 34.72 | Cytoplasm |
| *itfGA2ox7* | *itf09g05310.t1* | 9 | + | 2687278-2689298 | 348 | 6.28 | 39.72 | Cytoplasm |
| *itfGA2ox8* | *itf10g18170.t1* | 10 | - | 20149064-20150905 | 345 | 6.20 | 38.37 | Cytoplasm |
| *itfGA2ox9* | *itf10g20910.t1* | 10 | - | 22295683-22302772 | 354 | 6.28 | 40.61 | Cytoplasm |
| *itfGA2ox10* | *itf13g17820.t1* | 13 | - | 19736702-19739832 | 315 | 7.05 | 35.11 | Cytoplasm |
| *itfGA3ox1* | *itf03g19680.t1* | 3 | - | 15464418-15465827 | 363 | 5.09 | 41.25 | Cytoplasm |
| *itfGA3ox2* | *itf03g19690.t1* | 3 | - | 15473183-15474918 | 365 | 5.20 | 40.88 | Cytoplasm |
| *itfGA3ox3* | *itf03g19710.t1* | 3 | - | 15486971-15488562 | 362 | 5.55 | 40.86 | Cytoplasm |
| *itfGA3ox4* | *itf03g19720.t1* | 3 | - | 15490305-15492610 | 366 | 5.51 | 41.04 | Cytoplasm |
| *itfGA3ox5* | *itf03g19770.t1* | 3 | + | 15550903-15552216 | 361 | 5.12 | 40.83 | Cytoplasm |
| *itfGA3ox6* | *itf03g19780.t1* | 3 | + | 15557945-15559125 | 364 | 5.79 | 41.30 | Cytoplasm |
| *itfGA3ox7* | *itf03g19790.t1* | 3 | + | 15573245-15574598 | 363 | 5.11 | 40.95 | Cytoplasm |
| *itfGA3ox8* | *itf03g19800.t1* | 3 | + | 15579866-15581042 | 361 | 5.35 | 41.20 | Cytoplasm |
| *itfGA3ox9* | *itf03g19810.t1* | 3 | + | 15587320-15588701 | 364 | 5.01 | 40.96 | Cytoplasm |
| *itfGA3ox10* | *itf03g19820.t1* | 3 | + | 15589911-15591822 | 364 | 5.64 | 40.70 | Cytoplasm |
| *itfGA3ox11* | *itf11g09620.t1* | 11 | + | 5673442-5675436 | 380 | 8.72 | 41.29 | Cytoplasm |
| *itfGA3ox12* | *itf15g22400.t1* | 15 | + | 21378524-21382600 | 344 | 7.88 | 38.06 | Cytoplasm |
| *itfGA20ox1* | *itf03g12980.t1* | 3 | + | 11214119-11215840 | 384 | 5.20 | 43.41 | Cytoplasm |
| *itfGA20ox2* | *itf05g14240.t1* | 5 | - | 17434432-17436650 | 386 | 6.00 | 43.54 | Cytoplasm |
| *itfGA20ox3* | *itf12g24950.t1* | 12 | - | 22542504-22544642 | 365 | 6.96 | 40.77 | Cytoplasm |
| *itbGA2ox1* | *itb01g19440.t1* | 1 | - | 25530313-25533665 | 334 | 6.02 | 37.23 | Cytoplasm |
| *itbGA2ox2* | *itb01g20350.t1* | 1 | + | 26604765-26606627 | 327 | 7.67 | 36.33 | Cytoplasm |
| *itbGA2ox3* | *itb02g09580.t1* | 2 | - | 6152445-6154666 | 337 | 6.76 | 37.51 | Cytoplasm |
| *itbGA2ox4* | *itb04g07280.t1* | 4 | - | 4892216-4896113 | 334 | 6.64 | 37.59 | Cytoplasm |
| *itbGA2ox5* | *itb04g23140.t1* | 4 | - | 28333315-28334259 | 314 | 9.19 | 34.72 | Cytoplasm |
| *itbGA2ox6* | *itb09g05760.t1* | 9 | + | 3287005-3288024 | 339 | 7.65 | 38.72 | Cytoplasm |
| *itbGA2ox7* | *itb10g04700.t1* | 10 | - | 4520045-4522387 | 314 | 5.71 | 35.11 | Cytoplasm |
| *itbGA2ox8* | *itb10g18480.t1* | 10 | - | 24465386-24467455 | 345 | 6.20 | 38.36 | Cytoplasm |
| *itbGA2ox9* | *itb10g21050.t1* | 10 | - | 26391899-26399658 | 353 | 6.33 | 40.57 | Cytoplasm |
| *itbGA3ox1* | *itb01g08610.t1* | 1 | - | 6929304-6930696 | 365 | 4.94 | 41.17 | Cytoplasm |
| *itbGA3ox2* | *itb03g20470.t1* | 3 | - | 18370493-18371742 | 367 | 6.36 | 42.28 | Cytoplasm |
| *itbGA3ox3* | *itb03g20480.t1* | 3 | - | 18378565-18380040 | 339 | 5.12 | 38.61 | Cytoplasm |
| *itbGA3ox4* | *itb03g20500.t1* | 3 | - | 18389327-18390702 | 364 | 5.14 | 41.14 | Cytoplasm |
| *itbGA3ox5* | *itb03g20510.t1* | 3 | - | 18404309-18405655 | 361 | 5.79 | 41.35 | Cytoplasm |
| *itbGA3ox6* | *itb03g20520.t1* | 3 | - | 18418480-18419987 | 362 | 4.99 | 40.48 | Cytoplasm |
| *itbGA3ox7* | *itb03g20580.t1* | 3 | + | 18487800-18491048 | 367 | 5.73 | 41.73 | Cytoplasm |
| *itbGA3ox8* | *itb03g20590.t1* | 3 | + | 18491909-18493940 | 311 | 5.68 | 34.88 | Cytoplasm |
| *itbGA3ox9* | *itb03g20600.t1* | 3 | + | 18496991-18498606 | 329 | 5.63 | 37.21 | Cytoplasm |
| *itbGA3ox10* | *itb11g10570.t1* | 11 | + | 7561956-7567246 | 412 | 8.44 | 44.95 | Cytoplasm |
| *itbGA3ox11* | *itb15g23360.t1* | 15 | + | 26175041-26179997 | 346 | 7.89 | 38.33 | Cytoplasm |
| *itbGA20ox1* | *itb03g13600.t1* | 3 | + | 13674112-13675782 | 384 | 5.32 | 43.38 | Cytoplasm |
| *itbGA20ox2* | *itb05g14850.t1* | 5 | - | 22151136-22153524 | 369 | 6.00 | 41.76 | Cytoplasm |
| *itbGA20ox3* | *itb12g25280.t1* | 12 | - | 26553845-26556076 | 364 | 6.75 | 40.70 | Cytoplasm |
