# Supplementary material for: Genome wide identification and expression analysis of gibberellin oxidase family genes in sweet potato and its two diploid relatives
Source: Sci Rep. 2026 Feb 1;16:6882. doi: 10.1038/s41598-026-37951-8 (PMC12916950; doi:10.1038/s41598-026-37951-8)
Supplement: Supplementary file 1 — Supplementary Information 1. [file 41598_2026_37951_MOESM1_ESM.zip › Supplementary materials/Table S2.docx]

**Table S2. The Conserved Motif Sequence of GAox genes Detected by MEME.**

| **MOTIF** | **WIDTH** | **BEST POSSIBLE MATCH** |
| --- | --- | --- |
| MOTIF 1 | 41 | KIKMACEEWGFFQVVNHGVPMELLAQIEHZARRFFDLPREE |
| MOTIF 2 | 29 | PISNAFVVNVGDLLQILSNGRFKSVLHRA |
| MOTIF 3 | 21 | GFGPHTDPSLJTLLYQSNTNG |
| MOTIF 4 | 50 | IVSSAHHRISVIYFFGPKLDVKISPPPKLIKDGDFPLYRPFTWKEYRKIK |
| MOTIF 5 | 29 | FCTVVEDYQEAMRGLAEKILELIFKSLGI |
| MOTIF 6 | 38 | KLLTLRSPDSPNGYGIIPISRFFNTLMWMEGFTLSGSP |
| MOTIF 7 | 15 | ESFLRLNSYPPCPDP |
| MOTIF 8 | 29 | TELDHLVPMDFKNVVQVPDTHTWLINNSS |
| MOTIF 9 | 33 | KEKAGPPNPFGYGNKKIGSNGDVGWVEYJLLSA |
| MOTIF 10 | 21 | HFDKALEVVRFNSVVVENANA |
