# Supplementary material for: Genome wide identification and expression analysis of gibberellin oxidase family genes in sweet potato and its two diploid relatives
Source: Sci Rep. 2026 Feb 1;16:6882. doi: 10.1038/s41598-026-37951-8 (PMC12916950; doi:10.1038/s41598-026-37951-8)
Supplement: Supplementary file 1 — Supplementary Information 1. [file 41598_2026_37951_MOESM1_ESM.zip › Supplementary materials/Table S4.docx]

**Table S4. Primers used in qRT‒PCR analysis.**

| **Gene ID** | **Primer sequence (5'-3')** |
| --- | --- |
| ibGA3ox8_F | TCCCGTAGCGCTTCTTGC |
| ibGA3ox8_R | ACTGCGAAATCCGTGGGG |
| ibGA3ox6_F | GTCGACGTGGAGCCCATT |
| ibGA3ox6_R | GGCCCGATGTAACACGCT |
| ibGA2ox4_F | GTTGGATCCCACGGCCAA |
| ibGA2ox4_R | GGGACGCCATGGTTGACA |
| ibGA20ox4_F | CCGCCACGAGTCCAACAT |
| ibGA20ox4_R | GAAACGTCCGCAATGGCC |
| ibGA20ox1_F | GGAGTTGACGCGGACCTC |
| ibGA20ox1_R | GCTCGCCCAGCTTTCTGA |
| ibGA2ox1_F | ATCCTACCCTCCGGCGTT |
| ibGA2ox1_R | TTCCGCCGCCTCCATTTC |
| ibGA2ox8_F | CGCCAAACACCTCCTCGT |
| ibGA2ox8_R | TGAGGGCCTCGGATTCGA |
| ibGA2ox6_F | ATGACACCACAGGCCTGC |
| ibGA2ox6_R | ACCTGCAGGGAGTCACCA |
| ibGA3ox7_F | GGATCGCCGTTGGAGCTT |
| ibGA3ox7_R | GCCAGGCCCCTCATTTGT |
| ibGA3ox5_F | GGCCCCACACAGAGACAC |
| ibGA3ox5_R | GCTCCACGTCGACCCATT |
| ibGA3ox3_F | CGTCAACGTCAGCGACCT |
| ibGA3ox3_R | ACTCGCTCACAATGGCCC |
| ibGA2ox5_F | GTTGGATCCCACGGCCAA |
| ibGA2ox5_R | GGCTTCCAAGGGGACACC |
| ibGA20ox3_F | ACTCGCCGTCCCTCTCAT |
| ibGA20ox3_R | GTGGTTGGCGACGAGGAA |
| ibGA2ox2_F | CCAGTTCCTGCTGACCCG |
| ibGA2ox2_R | GAACAGCTCCCCACCACC |
| ibGA2ox9_F | CGTCAAAGCCTGCGAGGA |
| ibGA2ox9_R | GGGCCGGCGTTTTCTTTG |
| ibGA2ox10_F | GAAGTGGGCGGGCTACAA |
| ibGA2ox10_R | AGCCTGCAACATGTCCCC |
| ibactin_F | AGCAGCATGAAGATTAAGGTTGTAGCAC |
| ibactin_R | TGGAAAATTAGAAGCACTTCCTGTGAAC |
