# Supplementary material for: Genome wide identification and expression analysis of gibberellin oxidase family genes in sweet potato and its two diploid relatives
Source: Sci Rep. 2026 Feb 1;16:6882. doi: 10.1038/s41598-026-37951-8 (PMC12916950; doi:10.1038/s41598-026-37951-8)
Supplement: Supplementary file 1 — Supplementary Information 1. [file 41598_2026_37951_MOESM1_ESM.zip › Supplementary materials/Table S5.docx]

**Table S5. Primers used in subcellular localization.**

| **Gene ID** | **Primer sequence (5'-3')** |
| --- | --- |
| ibGA20ox1_GFP_F | ACAAATCTATCTCTCTCGAGATGGCGATTGAATGTATG |
| ibGA20ox1_GFP_R | GCTCACCATGGATCCTTGCCATTGCCGCTGCT |
| ibGA3ox8_GFP_F | ACAAATCTATCTCTCTCGAGATGAACCAAGAAATC |
| ibGA3ox8_GFP_R | GCTCACCATGGATCCTATTGCTTCCACTTCGCT |
